# Unique Attributes of the Laurel Wilt Fungal Pathogen, *Raffaelea lauricola*, as Revealed by Metabolic Profiling

**DOI:** 10.3390/pathogens10050528

**Published:** 2021-04-27

**Authors:** Ross Joseph, Michelle Lasa, Yonghong Zhou, Nemat O. Keyhani

**Affiliations:** 1 Department of Microbiology and Cell Science, University of Florida, Gainesville, FL 32611, USA; josephr1@ufl.edu (R.J.); mlasa@ufl.edu (M.L.); zyh800623@utibet.edu.cn (Y.Z.); 2 College of Science, Research Center for Qinghai-Tibet Plateau Ecology, Tibet University, Lhasa 850000, China

**Keywords:** *Raffaelea lauricola*, laurel wilt, beetle symbiont, phenome, metabolism, chemical sensitivity, fatty acids, lipid droplets, lipid dyes

## Abstract

*Raffaelea lauricola* is the causative agent of laurel wilt, a devastating disease of lauraceous trees. *R. lauricola* is also an obligate nutritional symbiont of several ambrosia beetle species who act as vectors for the pathogen. Here, we sought to establish the baseline “phenome” of *R. lauricola* with knowledge concerning its metabolic capability, expanding our understanding of how these processes are impacted by environmental and host nutrients. Phenotypic screening using a microarray of over one thousand compounds was used to generate a detailed profile of *R. lauricola* substrate utilization and chemical sensitivity. These data revealed (i) relatively restricted carbon utilization, (ii) broad sulfur and phosphate utilization, and (iii) pH and osmotic sensitivities that could be rescued by specific compounds. Additional growth profiling on fatty acids revealed toxicity on C10 substrates and lower, with robust growth on C12–C18 fatty acids. Conditions for lipid droplet (LD) visualization and LD dynamics were examined using a series of lipid dyes. These data provide unique insights regarding *R. lauricola* metabolism and physiology, and identify distinct patterns of substrate usage and sensitivity which likely reflect important aspects of the host-microbe interface and can be exploited for the development of strategies for mitigating the spread of laurel wilt.

## 1. Introduction

Laurel wilt is a lethal disease of susceptible plants in the family Lauraceae, a group containing over 600 species including avocado, red bay, swamp bay, and sassafras [1]. This disease is now endemic in the southeastern United States, where it is caused by the invasive ambrosia fungus, *Raffaelea lauricola* (Ascomycota: Ophiostomatales). The fungus is vectored to host plants by at least 10 different species of native and invasive ambrosia beetles within specialized fungal transport organs termed mycangia [2,3]. Beetle vectors burrow into the sapwood of lauraceous hosts, excavating galleries where they raise their brood and farm their symbiotic ambrosia fungus partner as their sole food source (ambrosia symbiosis) [4,5]. Upon exposure to *R. lauricola*, infected trees exhibit rapid wilting of terminal leaves and branches, leading to the death of part or all of the tree [6,7]. Wilting is preceded by the development of internal symptoms that include tylose and gel deposition, which impede water transport through blocked xylem vessel elements [8]. This disease has advanced throughout the southeastern United States largely unchecked and is responsible for the death of over 500 million trees since its introduction less than two decades ago. In 2012, laurel wilt was first detected in the avocado growing region of Florida, thus threatening a multi-billion-dollar industry with yearly projections of up to 50–60 million dollars in losses [9,10]. Although the exact mechanisms of pathogenesis exerted by this fungus on host trees remain obscure, during invasion, fungal cells must overcome host defenses by detoxifying plant defense compounds and locating suitable carbon sources for growth. Some fungi, and potentially *R. lauricola*, may have the capability to subsist on the gel contents deposited by plants within xylem vessels [11,12,13]. An analysis of the gel contents of laurel wilt-infected trees revealed that their primary components were pectins, phenolics, and lipids, suggesting that detoxification of phenolics and assimilation and utilization of lipids and other host carbon sources may be important strategies for fungal growth and survival within host trees [8]. 

Additionally, fungal cells contained within the mycangia of beetle hosts must subsist on secretions, which have been described as oily, waxy, and slightly acidic, from glandular cells in or near these symbiotic organs during dispersal to new host trees [14,15]. Ambrosia beetle mycangia have been shown to selectively promote the active growth of symbiotic ambrosia fungi, while suppressing the growth of non-symbiotic or contaminating fungi, suggesting an as-of-yet undescribed mechanism by which these organs maintain cultivar purity across beetle generations [16]. To date, the glandular secretions from these organs remain poorly characterized; however, the mycangium lumen into which they flow has been demonstrated to contain fatty acids, phospholipids, free sterols, sterol esters, triglycerides, and an abundance of amino acids including alanine, valine, and especially proline [15]. It is believed that the composition of these glandular secretions and luminal spaces may contribute to the sustained high-fidelity interaction between fungal symbiont and insect host [17]. As such, approaches that examine the metabolic potential of *R. lauricola* to utilize different substrates, as well as its cellular phenotype when grown on these substrates, could yield useful insights into the biology of this fungus and how it interacts with its hosts.

Phenotype microarrays are medium-throughput techniques that allow for the simultaneous characterization and monitoring of microbial cellular attributes under thousands of different conditions, including exposure to different drugs and chemicals, making them attractive methods for studying the metabolic capacity and cellular phenotypes of cells on a wide variety of substrates [18,19]. Tools for such “phenomic” characterizations include the Biolog (Hayward, CA, United States) phenotype microarray that can test for the presence or absence of a large range of specific cellular phenotypes, including the interrogation of metabolic pathways along with ionic, osmotic and pH effects. In addition, a detailed assessment of the utilization patterns of exogenous lipids with different chemical properties by fungi and their subsequent cellular phenotypes is warranted. Lipids, such as fatty acids, are important carbon sources for many fungi and, in some species, have been shown to represent an essential currency between symbiotic partners. Mycorrhizal fungi, for instance, are noted fatty acid auxotrophs and obtain these compounds from their plant host [20]. Fatty acids also play important roles in host sensing and signaling by pathogenic fungi. In the corn smut fungus, *Ustilago maydis*, growth on a triacylglyceride mixture containing corn oil induces a morphological switch from budding cells to filamentous growth, a switch associated with pathogenesis [21,22]. Fatty acid metabolites also affect the morphogenesis of the human pathogenic fungus, *Candida albicans*, and metabolites of fatty acids can be used as quorum sensing molecules by this fungus [23,24]. 

Once incorporated into cells, fatty acids are converted to neutral lipids in the endoplasmic reticulum and stored in organelles termed lipid droplets [25]. Lipid droplets (LD) are unique cellular organelles consisting of a neutral lipid core made primarily of triacylglycerols (TAG) and sterol esters (SE) and enclosed by a monolayer of phospholipids, distinguishing them from other organelles that have aqueous cores and phospholipid bilayers [26,27]. LDs contain dozens of different proteins on their surfaces, form tight associations with other organelles and cellular structures, and play essential roles in diverse cellular processes such as energy homeostasis, oxidative stress, detoxification, membrane stability, and gene regulation [28,29,30,31,32]. A number of bacterial and fungal pathogens use their own lipid droplets, or exploit host lipid droplets, as a means of initiating and maintaining infection [33,34,35]. The rice blast fungus, *Magnaporthe grisea*, for instance, mobilizes lipid droplets during infection structure (appressoria) formation, where they are degraded for use as energy stores and for maintaining the extremely high turgor pressure necessary to infect its host by mechanical penetration of tissues [36,37]. Lipid droplets also aid fungi in sequestering and thereby overcoming the toxic effects of exogenous and endogenous lipophilic toxins, which may play a key role in overcoming host defenses, as well as maintaining self-resistance to endogenous toxic compounds stored in fungal cells [38] 

A number of studies have examined the fungicide sensitivities of *R. lauricola*, and temperature and pH profiling indicated unique features of the fungus that include cold adaptation and poor growth at pH > 7.5 [39,40,41]. Here, using phenotyping arrays, we significantly expand characterization of the *R. lauricola* phenome to include a broad range of substrate utilization responses. In addition, we performed: (a) an examination of the dynamic range of *R. lauricola* fatty acid utilization, (2) a comparative analysis of LD visualization methods for assessing LD dynamics, and (c) characterizing the effects of fatty acids on LD formation. These data provide a critical baseline phenome for *Raffaelea lauricola* and provide tools for the assessment of fatty acid and LD formation in the growth and development of the fungus during saprophytic growth, symbiotic association (with beetles), and/or tree infection. 

## 2. Results

### 2.1. Phenotype Microarrays

**Figure 1:**
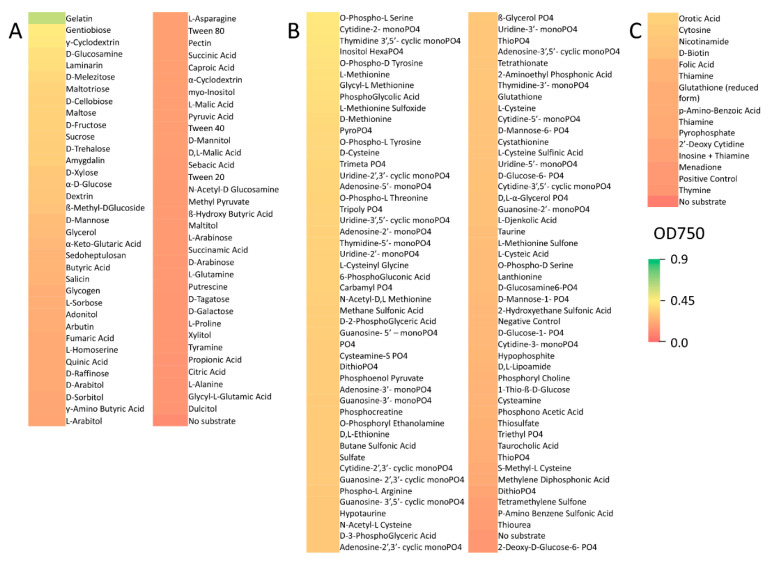
Heat map of substrates capable of supporting poor (0.15–0.3), moderate (0.3–0.6), and robust (>0.6) growth by *R. lauricola*. (**A**) Carbon substrates, (**B**) phosphorus and sulfur substrates, and (**C**) nutrient supplements (e.g., cofactors, vitamins, nucleotides, etc.).

Phenotype microarrays were performed as detailed in the Methods section and a summary of the results is presented in Table 1. For the purposes of comparative analyses, substrates were divided into four categories on the basis of growth as determined by OD_750_ readings as follows: (1) little to no growth: OD_750_ = 0–0.15, (2) poor growth: OD_750_ = 0.15–0.3, (3) moderate growth: OD_750_ = 0.3–0.6, and (4) robust growth: OD_750_ > 0.6). Amongst the 190 carbon sources tested, *R. lauricola* showed little to no growth on 122 (64%) of these substrates (Supplemental Figure S1, Note: in all instances compounds and/or conditions that resulted in little to no growth are listed in the Supplemental data section). These compounds included acetoacetic acid, sorbic acid, and both d and l galactonic acid-ƴ-lactone. *R. lauricola* showed poor growth on 51 carbon sources (27%), including pectin, d and l arabitol, and salicin; moderate growth on 16 substrates (8%), including gentiobiose, ƴ-cyclodextrin, and d-glucosamine, and robust growth on only 1 compound (0.5%), gelatin (Figure 1).

**Figure 2:**
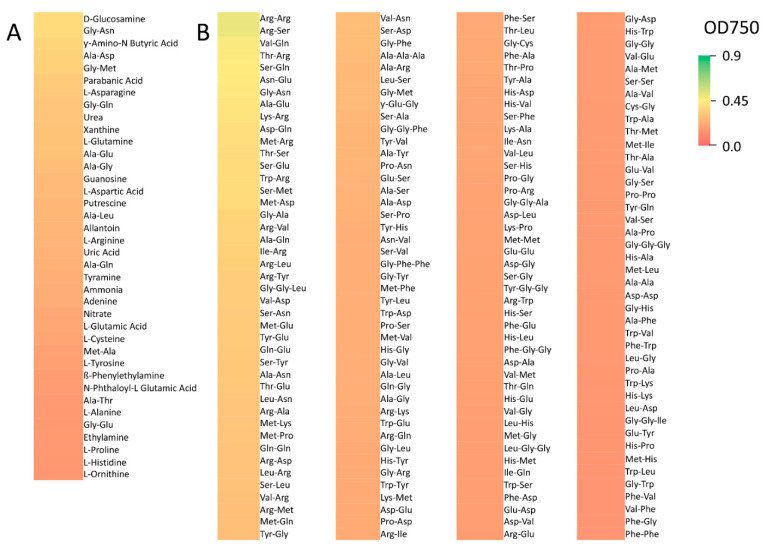
Heat map of nitrogen substrates which supported poor to robust growth. (**A**) simple nitrogen substrates and (**B**) peptide nitrogen substrates.

*R. lauricola* showed little to no growth on 57 (60%) out of 95 simple nitrogen sources tested (Supplemental Figure S3). These substrates included histamine, d-serine, and d-lysine. *R. lauricola* showed poor growth on 26 compounds (27%), including ammonia, nitrate, and l-arginine, and moderate growth on 12 substrates (13%), including d-glucosamine, and the dipeptides, gly-asn, and ala-asp (Figure 2). No robust growth was noted for any of the simple nitrogen substrates tested. Amongst an additional 282 peptide nitrogen sources tested, *R. lauricola* showed little to no growth on 114 (40%) of these, including val-tyr-val, pro-hyp (hydroxy proline), and asp-trp (Supplemental Figure S3). *R. lauricola* showed poor growth on 129 compounds (46%), including arg-met, leu-ser, and ser-asp, moderate growth on 39 substrates (14%), including arg-ser, thr-arg, and arg-arg. No robust growth was noted on any of these peptide nitrogen sources tested. *R. lauricola* was capable of at least poor growth on all of the 94 phosphorus and sulfur sources tested. Poor growth was seen on 28 compounds (30%), including thiophosphate, *S*-methyl-l-cysteine, and tetramethylene sulfone (Figure 1). *R. lauricola* showed moderate growth on the remaining 66 compounds (70%), including pyrophosphate, l and d-methionine, and inositol hexaphosphate. No robust growth was noted for any of the phosphorus and sulfur sources tested. An additional 94 different nutrient supplements were tested as potential growth substrates, and *R. lauricola* showed little to no growth on 81 (86%) of these including l-proline, spermidine, and l-ornithine (Supplemental Figure S2). *R. lauricola* showed poor growth on 9 substrates (10%), including glutathione (reduced form), folic acid, and thiamine, and moderate growth on 4 compounds (4%), including nicotinamide, orotic acid, and cytosine. No robust growth was noted on any of the nutrient supplements tested (Figure 1).

**Figure 3:**
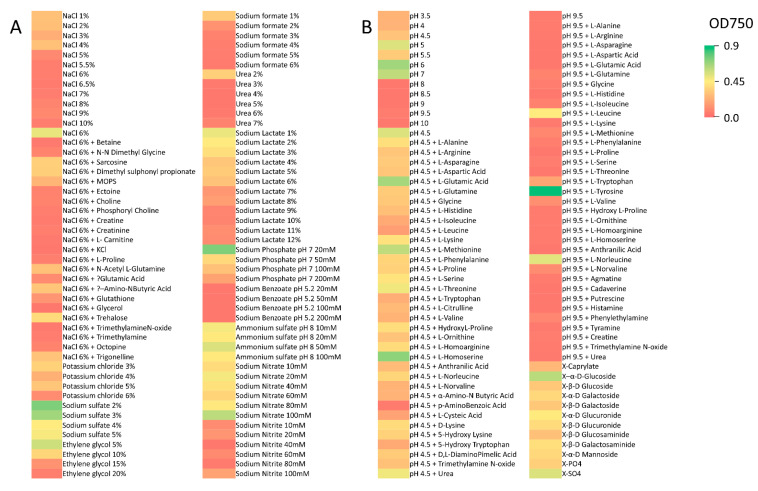
Heat map of all growth ranges (including no growth) for chemical compound/osmolytes tested, pH sensitivity assays, and X-linked substrates. (**A**) Chemical compound/osmolyte, and (**B**) pH conditions and X-linked substrates.

High sensitivity to a number of osmotic stress and denaturant conditions was noted for *R. lauricola*. Among 96 conditions tested, *R. lauricola* showed little to no growth under 48 (50%) conditions, including 5–10% NaCl, 2–6% sodium formate 2–6%, and 3–7% urea (Figure 3). *R. lauricola* showed poor growth on 12 substrates (13%), including 1–4% NaCl, 200 mM sodium phosphate pH 7, and 4% potassium chloride, moderate growth on 32 substrates (33%), including 6% NaCl + trehalose, 1–6% sodium lactate, and 10–80 mM sodium nitrate, and robust growth on 4 compounds (4%), including 100 mM sodium nitrate, 2–3% sodium sulfate, and 20 mM sodium phosphate pH 7. Interestingly, loss of growth in 6% NaCl was rescued by the addition of sarcosine, dimethyl sulfonyl propionate, MOPS, *N*-acetyl-l-glutamine, GABA, trehalose, or trigonelline (Figure 3). Amongst 96 different pH conditions tested, *R. lauricola* showed little to no growth on 39 (41%), including under alkaline conditions, i.e., pH 8–10, as well as in the presence of a number of supplements added at pH 9.5, and poor growth on 15 conditions (16%), including within the lower pH ranges (3.5 to 4) (Figure 3). *R. lauricola* showed moderate growth on 36 conditions (38%), including within the pH ranges 5–7. Robust growth was seen for 6 conditions (6%), including at low pH (pH 4.5) supplemented with l-homoserine or l-glutamic acid. Of note, the alkaline sensitivity of *R. lauricola* could be rescued by the addition of either l-leucine or l-norleucine (at pH 9.5). Under one condition, pH 9.5 + l-tyrosine, an artificially high reading was noted, as the substrate precipitated out of solution during incubation, forming an opaque layer at the bottom of the well. Within this panel of compounds, a series of 12 5-bromo-4-chloro-3-indoxyl- (X-) linked substrates were included. Cleavage of the X-moiety results in the release of the indicated carbon sources, and thus these substrates reflect enzymatic activities that include esterases (C8), α- and β-galactosidases, α- and β-glucuronidases, β-glucosamidases, β-galactosamindases, α-mannosidases, alkaline-phosphatases, and aryl-sulfatases. *R. lauricola* displayed from moderate to robust growth on all X-linked substrates, with the exception of X-caprylate (Figure 3).

**Figure 4:**
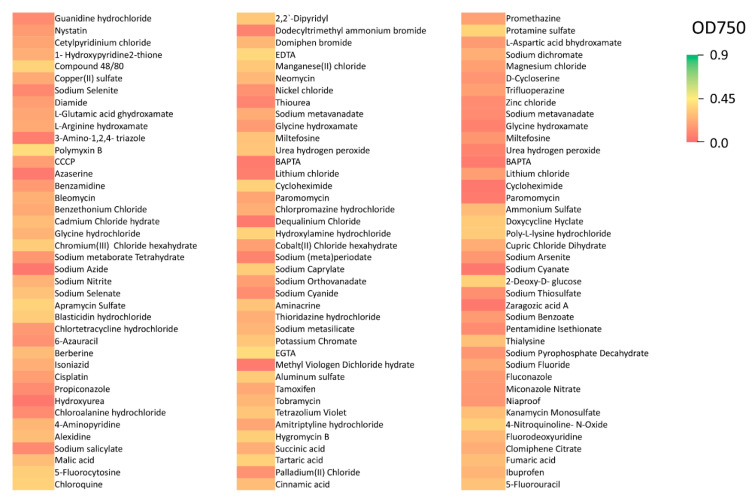
Heat map of growth on compounds testing chemical sensitivities.

Among the 120 chemicals tested in chemical sensitivity assays, *R. lauricola* showed little to no growth on 31 (26%) (Figure 4). These chemicals included sodium azide, sodium cyanate, and zaragozic acid A. *R. lauricola* showed poor growth on 59 compounds (49%) including in the presence of nystatin, malic acid, and tobramycin (Figure 4). Moderate fungal growth was seen on 30 compounds (25%) that included EDTA, hygromycin B, and chloroquine. 

### 2.2. R. Lauricola Fatty Acid Utilization

**Figure 5:**
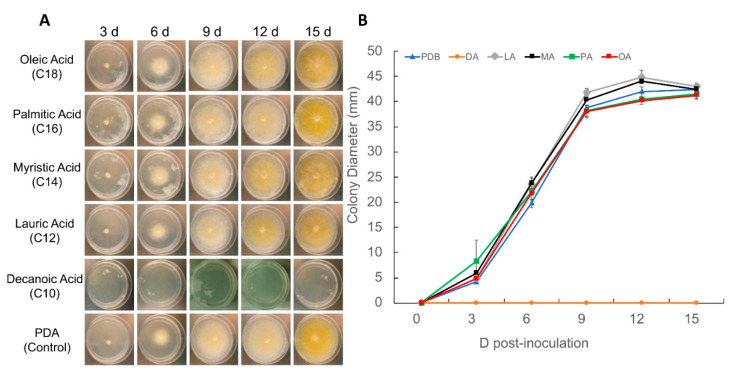
*R. lauricola* colony morphology and growth on control (PDA, potato dextrose agar, un-supplemented) plates, and plates supplemented with C10–C18 fatty acids as indicated. (**A**) Representative images of colony morphologies, and (**B**) growth curves based on colony diameter measurements. All experiments were performed using three technical replicates and the entire experiment repeated at least three times. Data presented ± SE (standard error).

Fatty acid utilization and mobilization has been linked to a wide variety of fungal pathogenic processes. The growth of *R. lauricola* on a range of fatty acids (at 0.05%) was examined in standard (PDA) media and in PDA media amended with 0.05% butyric acid (C4), hexanoic acid (C6), octanoic acid (C8), decanoic acid (C10), lauric acid (C12), myristic acid (C14), palmitic acid (C16), and oleic acid (C18) (Figure 5). Fatty acids below chain length C12 completely inhibited the growth of *R. lauricola,* whereas the fungus showed robust growth on fatty acids C12–C18. However, a number of differences in colony morphology were noted on the different fatty acids. On standard PDA media, fungal colonies formed as a thick mat of mycelia at the inoculation point that spread and become thinner and more web-like as growth emanated towards the edges of the plates (Figure 5). Colonies were also noted to begin as a tan color and darken to a yellow color with age (15 d incubation). Compared to the standard PDA plate, colonies growing in the presence of oleic, myristic, or lauric acid became pigmented earlier (12 d), whereas pigmentation in plates containing palmitic acid occurred as on standard media (15 d); however, at this time, these colonies appeared to be the most brightly pigmented amongst the conditions tested.

### 2.3. Assessment of Fluorescent Lipid Dyes for Lipid Droplet (LD) Visualization

In order to further probe the dynamics of lipid utilization, a total of five different lipid dyes: Lipi-Blue, Lipi-Red, Lipi-Green, Nile Red and BODIPY were evaluated to determine their relative efficacies in visualizing lipid droplet (LD) physiology in *R. lauricola.* Fungal cells were grown over a 24-h time course in standard liquid media (potato dextrose broth, PDB) and PDB supplemented with 0.05% oleic acid and aliquots were taken and stained with the various dyes as detailed in the Methods section. Representative images of *R. lauricola* cells grown in PDB and stained with the Lipi-dyes; i.e., either Lipi-Red, Lipi-Blue, or Lipi-Green revealed weak staining of initially germinating (12 h) *R. lauricola* conidia with the Lipi-Green and Lipi-Red dyes, and a somewhat clearer staining of punctate (LD) structures within the cells when using the Lipi-Blue dye (Supplemental Figure S4). Both an increase in intensity and overall staining of LDs could be seen over the examined time course of cell (hyphal) growth, i.e., at the 16, 20, and 24 h time points using Lipi-Red. Less consistent results were seen using the Lipi-Green and Lipi-Blue dyes (Supplemental Figure S4). Staining of *R. lauricola* cells grown in PDB supplemented with 0.05% oleic acid, showed a marked increase in staining using Lipi-Red, particularly at the later time point. Similarly, Lipi-Green staining was increased and LDs easier to visualize in PDB + oleic acid grown cells, whereas inconsistent/no clear increases were seen using the Lipi-Blue dye. 

**Figure 6:**
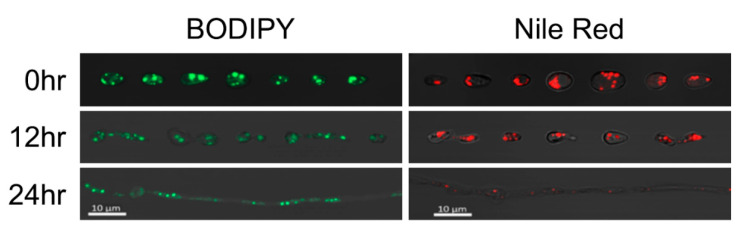
Representative images of lipid droplet (LD) staining in *R. lauricola*; control (PDB) LDs stained with BODIPY (left) and Nile Red (right) at 0 h—(spores), 12 h—(germinating spores), and 24 h—(hyphal growth) h timepoints in indicated media.

Both BODIPY and Nile Red showed clear staining of large globules in *R. lauricola* conidia harvested from PDA plates (Figure 6 and Supplemental Figure S5). Subsequent growth in PDB and PDB amended with 0.05% oleic acid revealed conidium and emerging germ tubes (12 h time point) and discrete punctate staining of LDs in growing hyphae. Signals were enhanced 2–6 fold over a similar time course of *R. lauricola* cells grown in PDB supplemented with oleic acid as compared to unamended PDB, with Nile Red showing a clearer increase in response over the time course examined. Quantification of the observed fluorescent signals was performed for samples stained with the BODIPY and Nile Red dyes, and these data revealed higher fluorescence in oleic acid treatment groups as compared to control (PDB) groups (Supplemental Figure S5). Nile Red fluorescence was initially similar between control and supplemented groups during early timepoints; however, fluorescence intensity increased dramatically in the oleic acid-treated group whereas the intensity remained relatively stable in the control group. BODIPY fluorescence was observed to be consistently higher in the oleic acid-treated group compared to the control group across all timepoints, although to a lesser degree than Nile Red. Based on these results, we selected Nile Red and BODIPY as the best candidate lipid dyes for use in further LD visualization experiments.

### 2.4. Lipid Droplet Physiology: Effect of Different Fatty Acids

**Figure 7:**
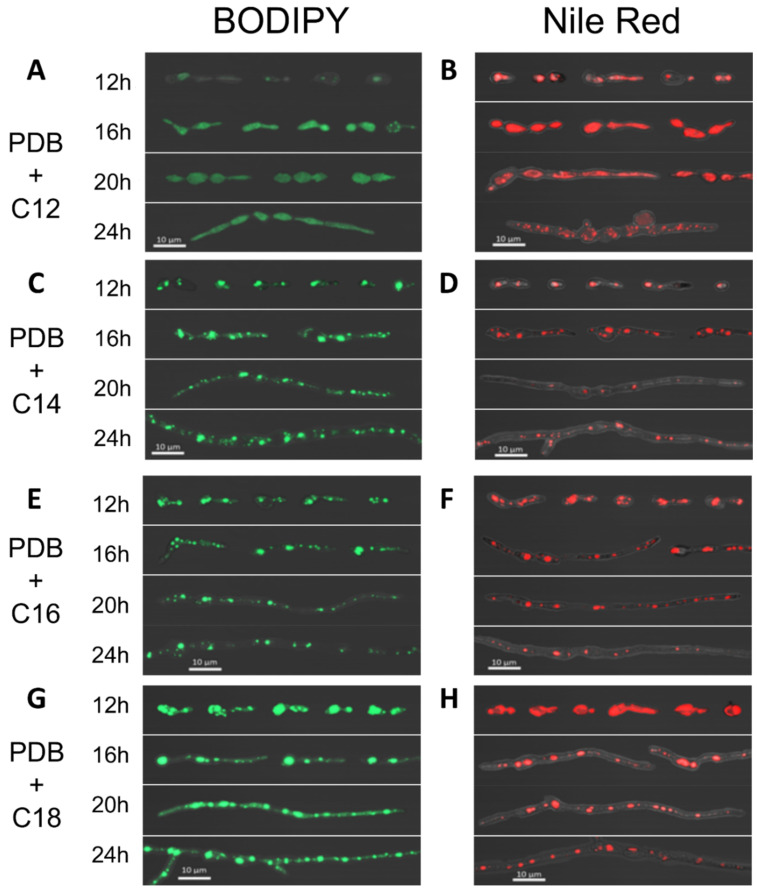
Representative images of staining of *R. lauricola* cells using BODIPY and Nile Red during growth in PDB (potato dextrose broth) + 0.05% C12 fatty acid (**A**,**B**), C14 (**C**,**D**), C16 (**E**,**F**), and C18 (**G**,**H**) over the indicated time course.

As BODIPY and Nile Red were demonstrated to give the most consistent results in terms of LD staining in *R. lauricola*, the analyses of LD formation was extended to growth on other fatty acids including lauric, myristic, and palmitic acids. Fungal cells were grown on the indicated fatty acids (0.05% and including unamended PDB and PDB + oleic acid) and aliquots of cells harvested over a time course (12–24 h) and stained with either BODIPY or Nile Red (Figure 7). As compared to LD physiology in un-supplemented media, a number of different LD phenotypes were observed: (1) strong induction of LD formation by oleic acid, as noted by larger and brighter punctate spots of fluorescence distributed across cells, (2) intermediate induction of LDs in myristic and palmitic acids, and (3) diffuse, less intense, (BIODIPY or Nile Red) signals, that were evenly distributed across the entire length of most conidia and hyphae in the presence of the shortest fatty acid chain length tested (that allowed for growth), namely lauric acid (C12). Furthermore, growth appeared stunted in the presence of lauric acid, with shorter swollen/bulging hyphae noticeable. Comparisons between Nile Red and BODIPY-stained samples within corresponding treatment groups revealed generally comparable LD distribution and size/shape; however, in the lauric acid-grown samples, diffuse fluorescence gave way to punctate spots of fluorescence at later time points using Nile Red, but not with BODIPY, suggesting that the two dyes may have slightly different lipid affinities and thus may provide useful complementary information on lipid droplet physiology when used in parallel.

## 3. Discussion

Knowledge concerning the metabolic capability of a (fungal) pathogen can aid in our understanding of how the pathogenic process may be impacted by environmental and host nutrients that can be made available and used by the invading fungus. For instance, an important virulence attribute of the human pathogenic fungus *Candida albicans* is its ability to scavenge oligopeptides and amino acids from the host environment through expression of secreted aspartic proteases and a suite of dedicated oligopeptide transporters and amino acid permeases [42]. Zinc uptake was recently shown to play an important role in the fitness and pathogenicity of the fungus, *Blastomyces dermatiditis* [43]. Similarly, some plant pathogenic fungi such as *Botrytis cinerea* utilize the carbon compound mannitol to quench ROS-mediated plant defenses [44]. In the present study, we assessed the metabolic utilization patterns, chemical sensitivities, and corresponding cellular growth phenotypes of the laurel wilt pathogen and ambrosia beetle symbiont, *Raffaelea lauricola.*

Consistent with previous more limited studies [45,46], *R. lauricola* showed a restricted carbon utilization profile, with growth seen mainly on “simple” carbohydrates (e.g., glucose, galactose, fructose) and on di- and tri-, and polysaccharides. Intriguingly, the most robust growth was observed on the animal-based protein hydrolysate, gelatin, potentially due to its high nitrogen content [47]. Gelatin has been used as a model for plant cell wall-associated structural proteins, some of which are enriched in hydroxyproline and contain repetitive amino acid sequences [48]. Although speculative, gelatin-like peptides may also be found in the beetle host [49]. *R. lauricola* efficiently grew on many of the simple nitrogenous compounds presented, such as ammonia and nitrate, and particularly well on urea, putrescine (a simple polyamine), and d-glucosamine (an amino sugar). Growth was also improved on the nucleotides adenine and guanosine, and to a lesser extent guanine, but not on thymine or cytosine. A clear preference for growth on certain amino acids and short peptides was noted, including arginine, serine, glutamine, glutamate, and methionine. The finding that methionine, when presented within short peptides, can support strong growth by *R. lauricola* is somewhat surprising given that this amino acid was not utilized efficiently by Ophiostomataceae species [46], although methionine as a constituent of a small peptide was not directly investigated. This is further intriguing since some *Raffaelea* species may not be capable of synthesizing methionine themselves, suggesting a reliance on exogenous methionine [45]. Extracellular amino acid sensing and utilization impacts both basic cellular growth and fungal pathogenicity, with regulatory circuits such as the nitrogen catabolite repression (NCR), target of rapamycin (TOR) and SPS and transceptor-mediated amino acid sensing dedicated to perceiving and responding to extracellular amino acids [50]. As such, these utilization patterns may hold useful insights into how *R. lauricola* is able to establish and maintain infections in its plant hosts and/or form a symbiotic interaction with its insect partner. 

That many phosphorus and sulfur substrates improved growth is consistent with recent observations from transcriptomic data that alternative sulfur uptake and assimilation pathways are coordinately up-regulated during infection by *R. lauricola* in plant hosts [51], and lend evidence that sulfur metabolism may play an important role in *R. lauricola* infection and persistence in plant hosts. The more limited growth observed on substrates that act as nutrient supplements, e.g., orotic acid, cytosine, and nicotinamide, may suggest a relatively limited range of catabolic pathways by which *R. lauricola* can utilize such substrates, paralleling findings by other groups that have suggested that ambrosia fungi may lack many functional enzymes necessary for the degradation and/or utilization of substrates within biosynthetic pathways [45,52]. Indeed, *Raffaelea* species may lack genes encoding components needed to synthesize some essential vitamins, and these compounds may be supplied by bacteria and/or yeasts occupying the ambrosia microbiome [45]. In this light, the strong growth of *R. lauricola* on several vitamin supplements, including nicotinamide, d-biotin, and folic acid may support the idea of some degree of reliance on exogenous sources for these compounds. Conditions examining osmolyte parameters showed a clear preference for growth on sodium sulfate, sodium lactate, ammonium sulfate, and sodium nitrate by *R. lauricola.* Some of these compounds, such a nitrates and sulfates, would likely be encountered by *R. lauricola* in the sapwood of lauraceous species, as xylem elements form the main pathway for mineral nutrients to travel from root to shoot in plants [53,54]. Growth in media amended to different pH values confirmed the previously described growth sensitivity of *R. lauricola* to neutral/alkaline condition (poor growth at pH > 7, [39]). Intriguingly, our data revealed that the addition of certain amino acids (l-leucine and l-norleucine, and to lesser extents l-tryptophan and l-valine) could rescue the alkaline sensitivity even at pH 9.5. This may reflect the ability of *R. lauricola* to deaminate these compounds, thus releasing ammonia into the media and reducing the pH to acceptable levels. Similarly, *R. lauricola* was capable of growth at pH 8 in the presence of ammonium sulfate. Such results warrant further investigation regarding their relevance to *R. lauricola* growth under environmentally relevant conditions, and what role acidification of the environment may play in the pathogenesis and symbiosis of this organism. Reports describing the pH of sapwood in lauraceous plants under physiological conditions are largely absent; however, studies conducted in other herbaceous and woody plant species have found xylem pH values ranging between 5.5–6.5, and also that alkalization and acidification of xylem sap are important responses to a number of stressors including drought and impaired sap flow [55,56]. A number of amino acids, including leucine, have been reported from xylem sap, suggesting that these compounds may be encountered by *R. lauricola* during infection, and could potentially be utilized to alter the pH of the host environment [57]. Additionally, the observed growth on X-linked compounds (which test for the activities of specific enzymes), indicates that *R. lauricola* produces esterases, α- and β-galactosidases, α- and β-glucuronidases, β-glucosamidases, β-galactosamindases, α-mannosidases, alkaline-phosphatases, and aryl-sulfatases. With respect to X-caprylate, which showed poor growth in our assays, we cannot differentiate between the inability of this fungus to cleave the substrate and/or the potential toxicity of the resulting product (C8), which our data shows inhibits *R. lauricola* growth. *R. lauricola* was very sensitive to inhibitors of enzymes involved in essential biosynthetic pathways, e.g., azaserine, a structural analog of glutamine that inhibits enzymes involved in purine biosynthesis, hydroxyurea, which inhibits the enzyme ribonucleotide reductase, zaragozic acid, a squalene synthase inhibitor, and dequalinium chloride, whose multiple mechanisms of action include being a mitochondrial NADH:ubiquinone reductase inhibitor [58,59,60,61]. Interestingly, *R. lauricola* showed higher sensitivities to these compounds than to known selection agents for this fungus such as hygromycin B, which showed highly variable suppression of *R. lauricola* growth. 

In addition to carbon, nitrogen, phosphorus, and sulfur compounds, we assessed the ability of *R. lauricola* to grow on various fatty acids ranging from C4-C18. Fatty acid utilization has been shown to be critical for formation of infectious structures in a wide range of pathogenic fungi including animal, insect, and plant pathogens [23,33,62], and lipid utilization acts as a nexus mediating fungal virulence and responses to stress [63]. Complete inhibition of growth was seen for C4, C8, and C10 fatty acids, suggesting that the lower range of fatty acid chain lengths impose fungicidal or fungistatic effects on this species. Fungal inhibition by free fatty acids is a well-documented, with reports of many plant- and animal-pathogenic fungi such as *Botrytis cinerea, Fusarium oxysporum,* and *Candida albicans* by fatty acids of different chain length and degree of saturation [64,65,66,67]. Antifungal free fatty acids are typically believed to act by becoming incorporated into the fungal cell membrane, thus increasing membrane fluidity and leading to destabilization and cell death; however, many other cellular pathways may be involved in these mechanisms and remain to be uncovered [64]. Interestingly, the antifungal efficiency of fatty acids is generally considered to increase along with their chain length; however, for *R. lauricola*, we found that shorter chain-length fatty acids (chain-length C10 and below) provide the strongest antifungal activity, while the fungus is more capable of managing exposure to longer chain-length (> C12) fatty acids without experiencing significant alterations in growth rate. Thus, our data indicate that *R. lauricola* displays robust growth on fatty acids greater than C12, up until C18, the highest chain length tested, although some differences in overall colony morphology was noted on the different fatty acids. The ability of *R. lauricola* to cope with the presence of longer chain fatty acids may reflect an evolutionary strategy to survive within blocked xylem elements containing gels high in certain lipid species, although the lipid composition of laurel wilt-infected trees has not been investigated to our knowledge. 

As fatty acid utilization is directly linked to the formation of lipid droplets (LDs), a series of fluorescent lipid dyes including Nile Red, BODIPY, Lipi-Red, Lipi-Blue, and Lipi-Green were assessed for their ability to visualize LDs. Although all dyes were observed to stain lipid droplets in *R. lauricola* cells to differing degrees, the Lipi-Blue, -Green, and -Red dyes were seen to perform more poorly than Nile Red and BODIPY dyes, as indicated by qualitative confocal imagery. Lipi-Green showed only faint staining, which appeared more diffuse within the cell than the other dyes, especially at earlier time points. Lipi-Blue was also relatively faint; however, its localization within the cell appeared to correspond much more closely with LDs, especially at earlier time points. Lipi-Red staining of *R. lauricola* appeared very faint and diffuse in earlier time points, but image quality was seen to dramatically improve when using cells derived from later timepoints. By comparison, both Nile Red and BODIPY showed strong fluorescence localized almost entirely within structures consistent with being LDs across all time points and preliminary fluorescence quantification demonstrated a clear induction of LDs in oleic acid-treated samples compared to untreated samples. These observed differences in lipid droplet staining efficiency may be the result of differences in membrane permeability or lipid affinity between the different dyes, although further investigation may be necessary to confirm this. In some instances, e.g., cells grown in unamended media (PDB), Nile Red staining was considerably fainter at the 0 and 12 h time points. Fluorescence signals from Nile Red samples were also observed to fade much more rapidly than those stained with BODIPY, a result that is consistent with what has been reported by others [37,68,69]. BODIPY showed strong and consistent fluorescence across all time points observed as well as relatively low photobleaching qualities, making it a superior dye for use in *R. lauricola* lipid droplet studies. Fungal cells grown in the presence of exogenous fatty acids (PDB + C12–C18) displayed notable differences in LD numbers, size, and distribution as compared to cells grown under control (PDB) conditions. Oleic acid-treated cells were seen to accumulate a significant number of LDs as compared to untreated cells, whereas palmitic and myristic acid displayed varying patterns of LD accumulation that was intermediate between control and oleic acid-induced samples. Lauric acid-treated samples displayed the most notable differences in lipid mediated staining, as well as differences in cell morphology. *R. lauricola* grown on lauric acid appeared stunted in size, with bulging hyphae and with diffuse fluorescent staining using either Nile Red or BODIPY across the entire surface area of the cell, with few, if any, clear (punctate) LDs apparent. As fatty acids with chain length smaller than lauric acid (< C12) were toxic, it is likely that lauric acid is at the edge of toxicity for *R. laurciola*. Lipid buildup within the cell membrane, as has been suggested previously as a primary mechanism for fatty acid cytotoxicity in fungi, could explain the bulging appearance of lauric acid-treated cells, as well as their diffuse lipid staining [65,66]. Alternatively, fatty acids may also accumulate in the endoplasmic reticulum, where fatty acids are converted into phosphatidic acid (PA) before conversion to diacylglycerols (DAGs) and triacylglycerols (TAGs) for LD formation, giving the appearance of diffuse staining within the cell. Such a buildup of short-chain fatty acids within the ER may be the result of lauric acid not being utilized efficiently by enzymes responsible for conversion of lipids to PA (and subsequently to DAGs and TAGs), as many of these enzymes have been shown to have specific affinities for fatty acids with longer chain-lengths [64]. An inability to process these shorter chain-length fatty acids may, thus, be one potential explanation for their observed cytotoxicity on *R. lauricola* cells. The ability of Nile Red and BODIPY to show different lipid droplet physiologies for lauric acid-treated cells, especially in later time points, may provide clues to how this fatty acid is being processed and incorporated into *R. lauricola* cells, with future experiments targeting the mechanisms underlying the observed lipid droplet phenotype warranted.

In conclusion, we present an in depth metabolic and physiological profiling of the laurel wilt pathogen and ambrosia beetle symbiont, *R. lauricola*. These data can be used to complement existing genomic and transcriptomic datasets, allowing for genotype to phenotype observations of important metabolic and physiological traits such as substrate utilization, chemical sensitivities, and resistance to environmental stressors [51,70]. Coupled to more recent methods for the genetic manipulation of *R. lauricola* [41], specific hypotheses can be tested on the contributions of various physiological aspects of the fungus to virulence versus symbioses. Despite its varied lifestyles, our data show that the metabolic capacity of *R. lauricola* has certain restrictions, suggesting that specific mechanisms or pathways may be important for this fungus to persist in its disparate hosts. Fatty acid utilization and methods for LD visualization were developed that can be used to further probe the roles of these pathways in *R. lauricola* growth and development, and our data provides a foundation for dissecting the physiological parameters and requirements of *R. lauricola* as a plant pathogen and insect symbiont. 

## 4. Methods

### 4.1. Fungal Strains and Chemical Reagents

*R. lauricola* (CBS 127349, Centraalbureau voor Schimmelcultures Fungal Biodiversity Centre, Utrecht, The Netherlands) was kindly provided by R. Ploetz (UF-TREC, Homestead, FL, USA). The fungus was cultured on potato dextrose agar/broth (PDA/PDB) and Czapek-dox agar/broth (CZA/CZB) amended as indicated. Conidial suspensions were prepared by harvesting conidia from PDA plates after 7 d growth by flooding plates with 1–2 mL of sterile 0.05% Tween-20 solution. Cell suspensions were adjusted to desired concentrations after counting using a hemocytometer. Lipi-Red, Lipi-Green, and Lipi-Blue were obtained from Dojindo Molecular Technologies (Rockville, MD, USA). Nile Red, BODIPY, and fatty acids (decanoic, lauric, myristic, palmitic, and oleic acids) were purchased from Sigma-Aldrich (St. Louis, MO, USA).

### 4.2. Phenotype Microarrays

The Biolog phenotype microarray plate series (PM1, PM2A, PM3B, PM4A, PM5, PM6, PM7, PM8, PM9, PM10, PM21D, PM22D, PM23A, PM24C, PM25D, Biolog, Hayward, CA, USA) were inoculated according to the Biolog PM Procedures for Filamentous Fungi with slight modifications. Briefly, *R. lauricola* blastospores were harvested from a 3 d PDB cultures by filtration through sterile lens paper, followed by washing with sterile distilled water. Cells were washed once with sterile Filamentous Fungi-Inoculating Fluid (FF-IF, Biolog, catalogue # 72106) and finally resuspension in sterile FF-IF. The spore suspension was then used to inoculate 12 mL of sterile FF-IF to an OD_650_ = 0.1. This solution was then added to each of four inoculating fluid stock solutions (PM1.2, PM3,5–8, PM4, and PM9+). FF-IF was utilized for PM plates 1 and 2. FF-IF plus 100 mmol/L d-glucose, 5 mmol/L potassium phosphate (pH 6.0), and 2 mmol/L sodium sulfate was used for plates PM3, 5, 6, 7, and 8. FF-IF plus 100 mmol/L d-glucose was used for plate PM4. FF-IF plus yeast nitrogen base and 100 mmol/L d-glucose was used for plates PM9 and 10, and PM21–25. 100 μL of each inoculating fluid stock solution was then added to each of the wells in their respective plates, and wells were covered with sealing tape. Plates were incubated at 26 °C for 4 d, after which the endpoint OD_750_ was read for each well using a Tecan Infinite 200 Pro microplate reader. Each plate was run in duplicate, and endpoint OD_750_ readings for corresponding conditions were averaged across all data points and used to generate heatmaps of fungal growth in each condition. For chemical sensitivity plates, each condition was represented in quadruplicate and all replicates were averaged (8 total per condition). For comparisons of growth, raw OD_750_ readings from substrates and negative control wells were compared directly. 

### 4.3. Preparation of Lipid Droplet Dye Stocks and Working Solutions

Concentrated stock solutions of the fluorescent dyes were made in DMSO for Lipi-Blue, Lipi-Green, and Lipi-Red (1 mM), acetone for Nile Red (10 mM), and in PBS for BODIPY (5 mM) and stored at −20 °C until use. Working solutions of dyes were made fresh on the day of their intended use by diluting stock solutions in PBS to the desired concentration as follows: 0.1–0.5 μM for Lipi-Blue and Lipi-Green, 1–5 μM for Lipi-Red. 10 μM for Nile Red, and 1–5 μM for BODIPY. All dye solutions were maintained in the dark for a much as possible.

### 4.4. Fatty Acid Utilization and Lipid Droplet Staining

Fungal growth on PDA, and PDA plates supplemented with 0.05% of either decanoic acid, lauric acid, myristic acid, palmitic acid, or oleic acid (initially dissolved in hexane and added to the cooling media before pouring into Petri plates), were inoculated in the center of the plate with 5 μL of 5 × 10^8^ conidia/mL solution of *R. lauricola* cell suspension. Plates were incubated at 25 °C and growing colonies were photographed every 3 d over a 15 d growth period. Colony diameters were measured using the ImageJ software. Triplicate plates were examined for each condition, and the entire experiment replicated three times. 

Fungal growth and lipid assimilation were monitored in liquid cultures over time by inoculating 50 mL PDB or PDB supplemented with 0.05% lauric acid, myristic acid, palmitic acid, or oleic acid originally dissolved in 500 μL hexane) with 500 μL of 5 × 10^8^ spores/mL solution of *Raffaelea lauricola* conidia harvested from 7 d old PDA plates. Liquid cultures were grown at 25 °C with aeration (200 rpm orbital shaking) and cell suspension aliquots (5 mL) were collected for analysis at 12, 16, 20, and 24-h post-inoculation time points. Cells were harvested by centrifugation (4300 rpm for 5 min), and the fungal cells were washed 2× with PBS before being resuspended in 1 mL of the appropriate lipid dye working solution. Cells were then incubated in the dark at room temperature for 1 h, then washed 3× with PBS for microscopic visualization. A 500 μL aliquot of a 5 × 10^8^ spore solution (harvested from PDA plates) was also taken and processed directly as above with the appropriate lipid dye to generate a 0-h time point for growth. Preliminary quantification of BODIPY and Nile Red fluorescence was performed using the image analysis software imageJ (Supplemental Figure S5). Images were converted to 16-bit grey scale and the “freehand” tool was used to trace the outlines of individual fungal cells. The “analyze” tool, with “area integrated density” and “mean grey scale value” selected for “set measurement” parameters, was used to measure the selected area. Background readings were taken for normalizing fluorescence measurements to obtain a value for corrected total cell fluorescence. This process was performed for 120–160 cells per group in order to compare fluorescence between uninduced (PDB) and induced (PDB + Oleic acid) conditions.

### 4.5. Confocal Microscopy

Sample slides were imaged using a Zeiss lsm800 confocal microscope at 63× oil immersion with a differential interference contrast (DIC) filter with eight points of averaging and data collected using the Zen software. Filters used included: BODIPY; BodFl (Ex/Em 205/511), Nile Red; nilRe (Ex/Em 559/636), Lipi-Red; LTReN (Ex/Em 582/616), Lipi-Green; LTGrN (Ex/Em 498/507), Lipi-Blue; PacBl (Ex/Em 402/455). Bright-field images were also collected for all samples, and z-stacks of roughly 10–20 slices were recorded in order to capture fluorescence signals along hyphal strands spanning multiple planes of focus. Subsequent z-stacks were assembled into maximum intensity projections for figure images.

## Figures and Tables

**Table 1:** Summary of *R. lauricola* phenotype profiling.

Substrate/Compound Tested	Number Tested	Poor Growth (0.15–0.3) ^1^	Moderate Growth (0.3–0.6) ^1^	Robust Growth (>0.6) ^1^	Top Conditions and Sensitivities
carbon	190	51	16	1	Robust: gelatin, gentobiose, ƴ-cyclodextrin, d-glucosamine
simple nitrogen	95	26	12	0	Condition: gly-asn, ƴ-amino-*N* butyric acid, ala-asp
peptide nitrogen	282	129	39	0	Robust: arg-arg, arg-ser, val-gln, thr-arg
phosphorus and sulfur	94	29	66	0	Robust: *O*-phospho-l-serine, cytidine-2-monophosphate, thymidine 3′,5′-cyclic monophosphate, inositol hexaphosphate
nutrient supplements (e.g., cofactors, vitamins, nucleotides, etc.)	94	9	4	0	Robust: orotic acid, cytosine, nicotinamide, d-biotin
osmolytes	96	12	32	4	Robust: sodium sulfate 2%, sodium phosphate pH 7 20 mM, sodium sulfate 3%, sodium nitrate 100 mM Sensitive to: sodium benzoate 20–200 mM, urea 3–7%
pH conditions	96	15	36	6	Robust: pH 6–6.5, pH 4.5 + l-homoserine, pH 4.5 + l-glutamic acid Sensitive to: pH 8–10
X-linked compounds ^2^	12	1	10	1	Robust: X-α-d-glucoside Sensitive to: x-caprylate
chemical sensitivity	120	59	30	0	Robust: polymyxin B, EGTA, protamine sulfate Sensitive to: sodium cyanate, sodium azide, zargozic acid A

^1^ All readings reflect OD_750_ values, OD_750_ values < 0.15 were considered as essentially no growth. ^2^ X = 5-bromo-4-chloro-3-indoxyl.

## Data Availability

All data are provided in the manuscript and/or supplementary materials.

## Supplementary Materials

The following are available online at https://www.mdpi.com/article/10.3390/pathogens10050528/s1, Figure S1: Carbon compounds that supported little to no growth (OD_750_ = 0.0–0.15) by *R. lauricola*, Figure S2: Nutrient supplement compounds that supported little to no growth (OD_750_ = 0.0–0.15) by *R. lauricola*, Figure S3: Nitrogen compounds that supported little to no growth (OD_750_ = 0.0–0.15) by *R. lauricola*. A) Simple nitrogen compounds, and B) peptide nitrogen compounds, Figure S4: Assessment of the ability of lipid dyes, Lipi-Red (A), Lipi-Green (B), and Lipi-Blue (C) to stain lipid droplets in *R. lauricola*. Cells were grown in PDB (left) or in PDB + 0.05% oleic acid (C18, right), and sampled over a 12–24 h time course., Figure S5: Representative images of BODIPY and Nile Red staining lipid droplets (LD) in R. lauricola. (A) R. lauricola blastospores collected from PDA plates and immediately stained with BODIPY or Nile Red. (B) R. lauricola cells grown in PDB and sampled at 12-, 16-, 20-, and 24-h timepoints and stained using BODIPY and Nile Red dyes. (C) Nile Red staining comparison between PDB (control) and PDB + C18 (oleic acid) at 12-, 16-, 20-, and 24-h timepoints. (D) BODIPY staining comparison between PDB (control) and PDB + C18 (oleic acid) at 12-, 16-, 20-, and 24- h timepoints. (E) Quantification of Nile Red fluorescence intensity in cells grown under the indicated conditions. (F) Quantification of Nile Red fluorescence intensity in cells grown under the indicated conditions. At least 15–20 images were analyzed in three biological replicates for each time point/dye. Data are shown ± SE.
