# Supplementary material for: Unique Attributes of the Laurel Wilt Fungal Pathogen, Raffaelea lauricola, as Revealed by Metabolic Profiling
Source: Pathogens. 2021 Apr 27;10(5):528. doi: 10.3390/pathogens10050528 (PMC8146198; doi:10.3390/pathogens10050528)
Supplement: Supplementary file 1 [file pathogens-10-00528-s001.zip › pathogens-1187010-supplementary.pdf]

**Supplemental Materials**

# Insights from metabolic profiling of the laurel wilt fungal pathogen, *Raffaelea lauricola*

Ross Joseph[1], Michelle Lasa[1], Yonghong Zhou[1,2], Nemat Keyhani[1*]

[1]Department of Microbiology and Cell Science, University of Florida, Gainesville, Florida 32611, USA

[2]College of Science, Research Center for Qinghai-Tibet Plateau Ecology, Tibet University, Lhasa 850000, Tibet, PR China

Acetamide
δ-Amino Valeric Acid
Dihydroxy Acetone
L-Rhamnose
Mono Methyl Succinate
ß-Cyclodextrin
L-Arginine
D-Ribose
Bromo Succinic Acid
Palatinose
ß-Methyl-D Xyloside
Glycine
D-Malic Acid
Glycyl-L-Proline
α-D-Lactose
α-Methyl-D Glucoside
L-Alanyl-Glycine
1,2-Propanediol
Glycolic Acid
Lactulose
L-Histidine
α-Keto-Valeric Acid
Negative Control
Melibionic Acid
D-Fucose
D-Psicose
L-Fucose
4-Hydroxy Benzoic Acid
D-Lactic Acid Methyl Ester
Thymidine
ß-Methyl-D Galactoside
α-Methyl-D Mannoside
N-Acetyl-L Glutamic Acid
Inulin
i-Erythritol
Hydroxy-L Proline
L-Lactic Acid
L-Alaninamide
α-Methyl-D Galactoside
Stachyose
2,3-Butanediol

L-Leucine
D-Glucuronic Acid
D,L-α-Glycerol Phosphate
Citramalic Acid
D-Ribono-1,4-Lactone
D,L-Octopamine
Citraconic Acid
L-Isoleucine
L-Glutamic Acid
L-Lyxose
Malonic Acid
L-Glucose
D-Saccharic Acid
L-Lysine
D-Aspartic Acid
Phenylethylamine
D-Fructose-6- Phosphate
ß-D-Allose
N-Acetyl-ß-D Mannosamine
5-Keto-D Gluconic Acid
Uridine
Itaconic Acid
D-Tartaric Acid
α-Hydroxy Butyric Acid
D-Serine
L-Tartaric Acid
D-Galacturonic Acid
Oxalic Acid
Inosine
m-Hydroxy Phenyl Acetic Acid
D-Threonine
Glycolic Acid
L-Valine
N-Acetyl Neuraminic Acid
D-Alanine
α-Keto-Butyric Acid
Negative Control
2-Hydroxy Benzoic Acid
Tricarballylic Acid
Mannan
Adenosine

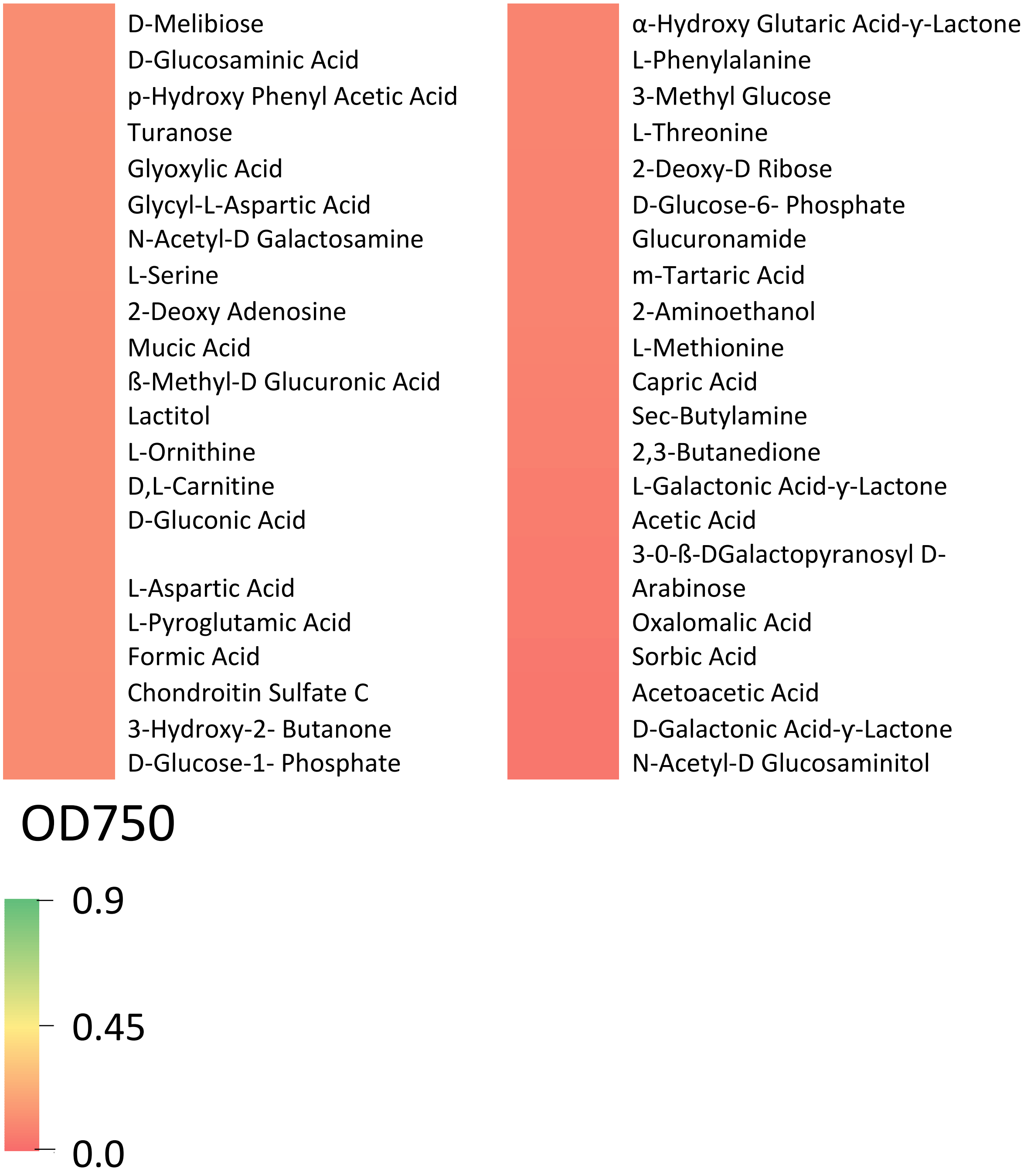


**Supplemental Figure S1.** Carbon compounds that supported little to no growth ($OD_{750}$ = 0.0-0.15) by *R. lauricola*.

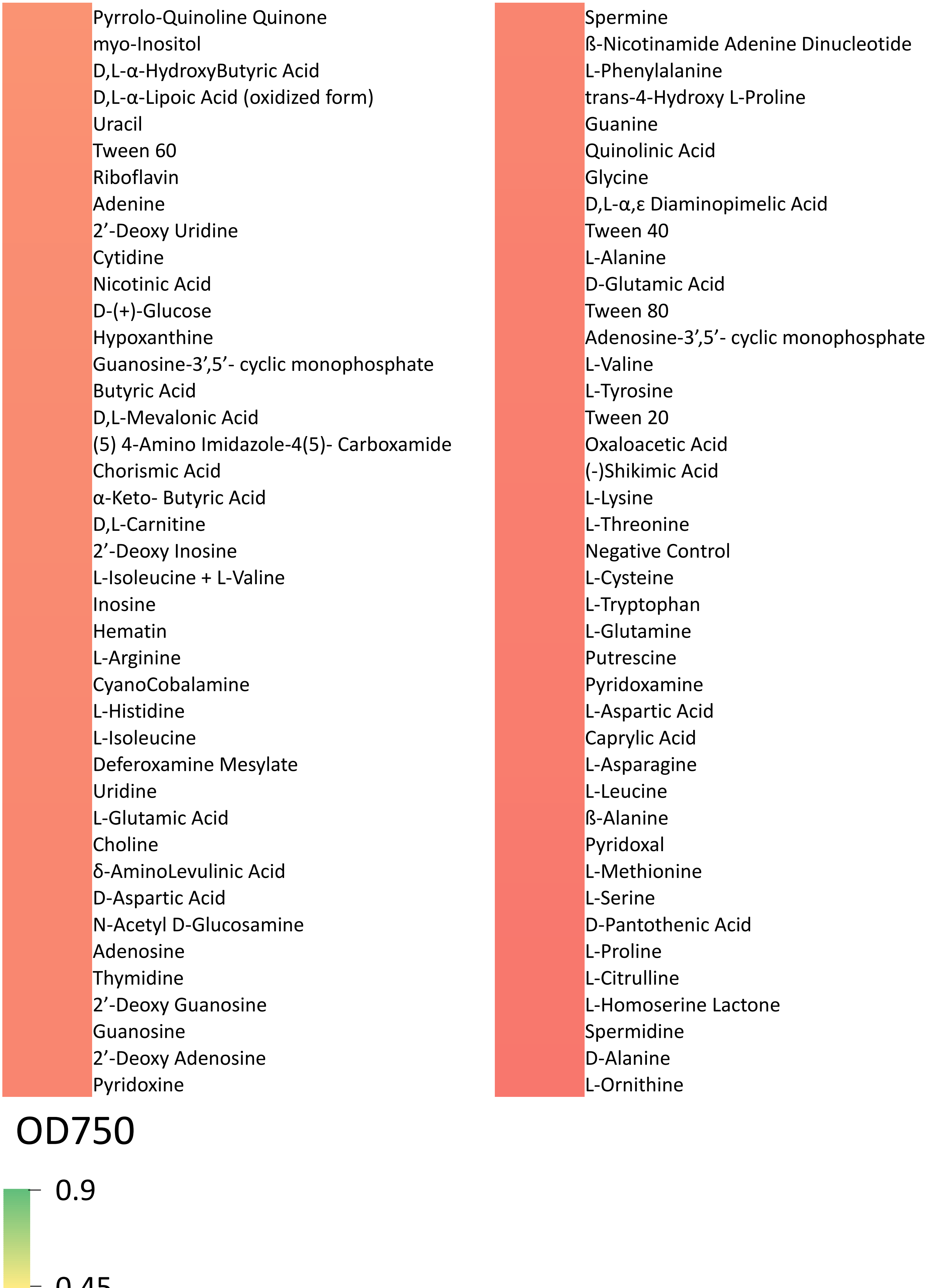
Pyrrolo-Quinoline Quinone
myo-Inositol
D,L-α-HydroxyButyric Acid
D,L-α-Lipoic Acid (oxidized form)
Uracil
Tween 60
Riboflavin
Adenine
2’-Deoxy Uridine
Cytidine
Nicotinic Acid
D-(+)-Glucose
Hypoxanthine
Guanosine-3’,5’- cyclic monophosphate
Butyric Acid
D,L-Mevalonic Acid
(5) 4-Amino Imidazole-4(5)- Carboxamide
Chorismic Acid
α-Keto- Butyric Acid
D,L-Carnitine
2’-Deoxy Inosine
L-Isoleucine + L-Valine
Inosine
Hematin
L-Arginine
CyanoCobalamine
L-Histidine
L-Isoleucine
Deferoxamine Mesylate
Uridine
L-Glutamic Acid
Choline
δ-AminoLevulinic Acid
D-Aspartic Acid
N-Acetyl D-Glucosamine
Adenosine
Thymidine
2’-Deoxy Guanosine
Guanosine
2’-Deoxy Adenosine
Pyridoxine
Spermine
ß-Nicotinamide Adenine Dinucleotide
L-Phenylalanine
trans-4-Hydroxy L-Proline
Guanine
Quinolinic Acid
Glycine
D,L-α,ε Diaminopimelic Acid
Tween 40
L-Alanine
D-Glutamic Acid
Tween 80
Adenosine-3’,5’- cyclic monophosphate
L-Valine
L-Tyrosine
Tween 20
Oxaloacetic Acid
(-)Shikimic Acid
L-Lysine
L-Threonine
Negative Control
L-Cysteine
L-Tryptophan
L-Glutamine
Putrescine
Pyridoxamine
L-Aspartic Acid
Caprylic Acid
L-Asparagine
L-Leucine
ß-Alanine
Pyridoxal
L-Methionine
L-Serine
D-Pantothenic Acid
L-Proline
L-Citrulline
L-Homoserine Lactone
Spermidine
D-Alanine
L-Ornithine
OD750
0.9

**Supplemental Figure S2.** Nutrient supplement compounds that supported little to no growth ($OD_{750}$ = 0.0-0.15) by *R. lauricola.*

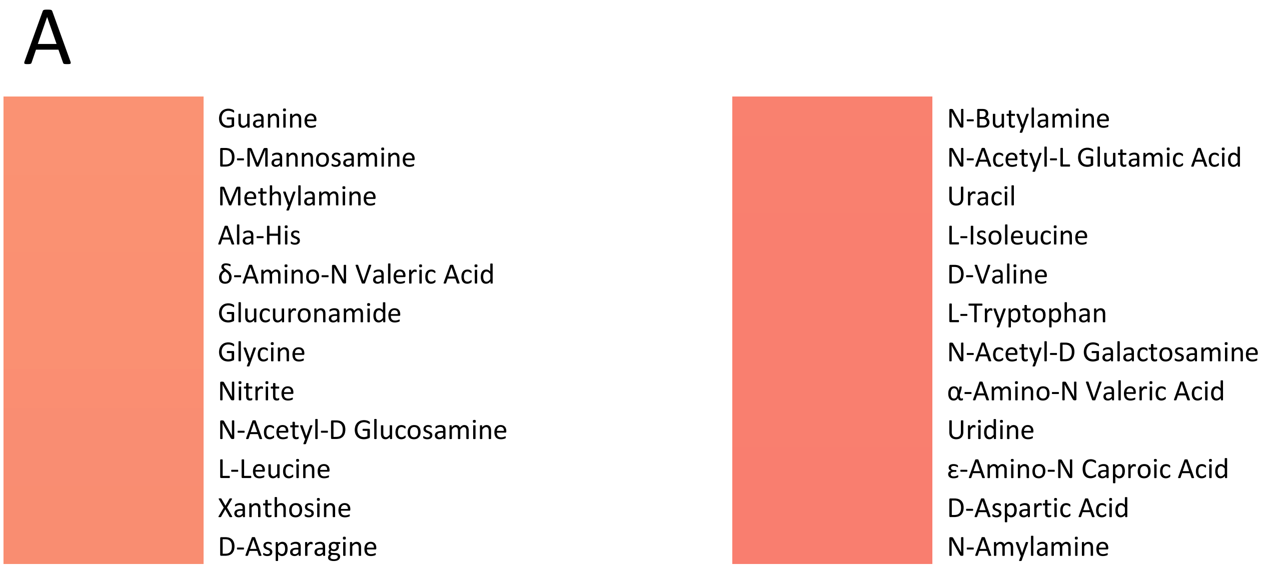

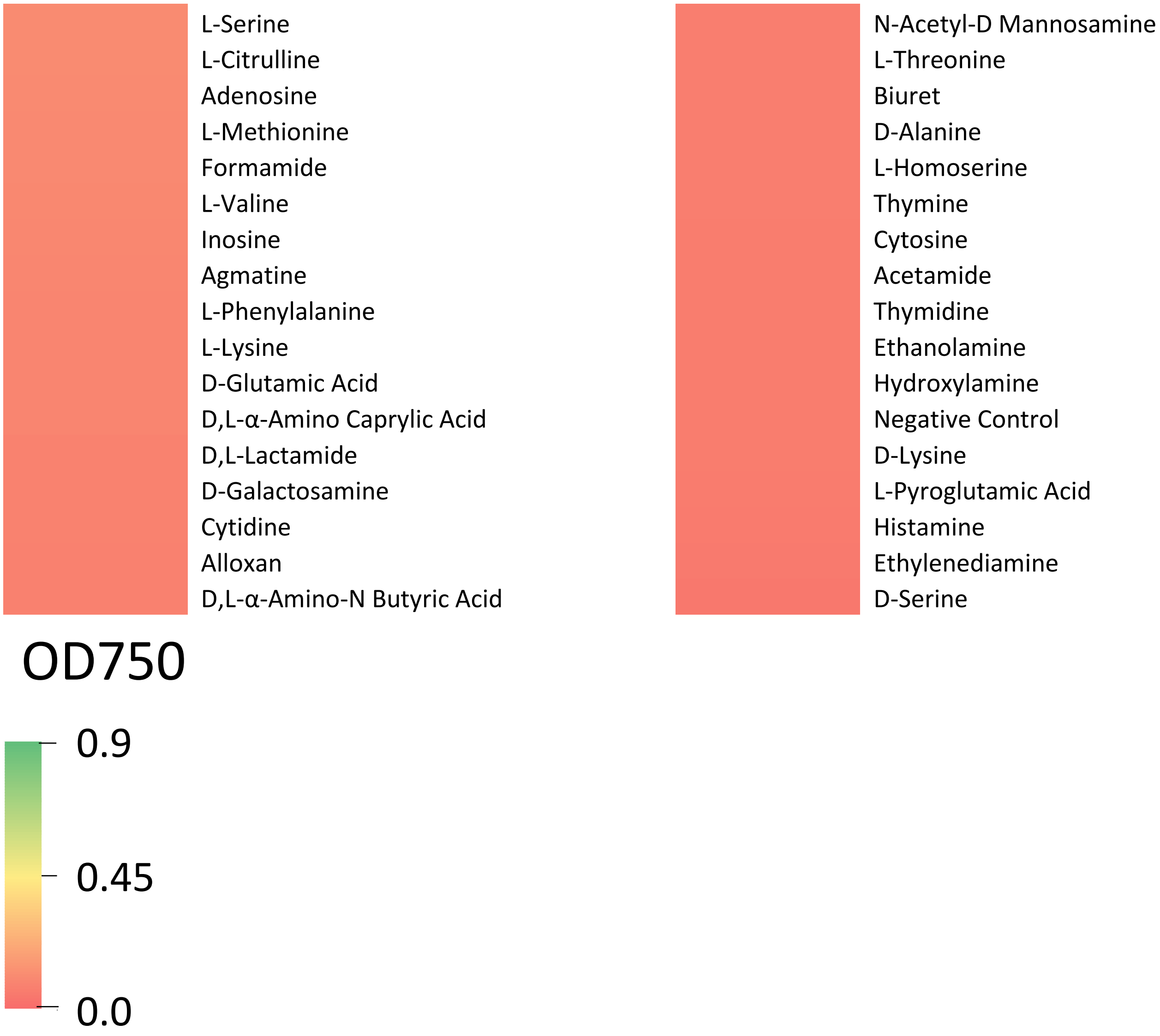
L-Serine
L-Citrulline
Adenosine
L-Methionine
Formamide
L-Valine
Inosine
Agmatine
L-Phenylalanine
L-Lysine
D-Glutamic Acid
D,L-α-Amino Caprylic Acid
D,L-Lactamide
D-Galactosamine
Cytidine
Alloxan
D,L-α-Amino-N Butyric Acid
N-Acetyl-D Mannosamine
L-Threonine
Biuret
D-Alanine
L-Homoserine
Thymine
Cytosine
Acetamide
Thymidine
Ethanolamine
Hydroxylamine
Negative Control
D-Lysine
L-Pyroglutamic Acid
Histamine
Ethylenediamine
D-Serine
OD750
0.9
0.45
0.0

B

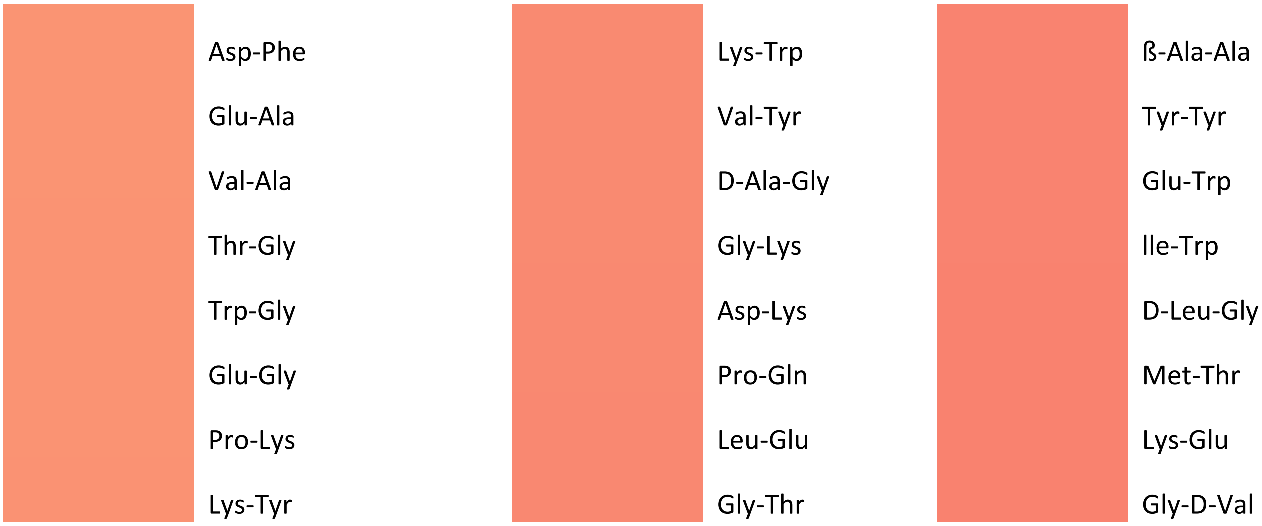
Asp-Phe
Glu-Ala
Val-Ala
Thr-Gly
Trp-Gly
Glu-Gly
Pro-Lys
Lys-Tyr
Lys-Trp
Val-Tyr
D-Ala-Gly
Gly-Lys
Asp-Lys
Pro-Gln
Leu-Glu
Gly-Thr
ß-Ala-Ala
Tyr-Tyr
Glu-Trp
Ile-Trp
D-Leu-Gly
Met-Thr
Lys-Glu
Gly-D-Val

Val-His
Ala-Lys
Ala-His
Phe-Tyr
Phe-Met
Lys-Phe
Ile-His
Leu-Tyr
Val-Lys
Leu-Val
Pro-Leu
Ile-Ser
Ile-Gly
Leu-Ala
Tyr-Phe
D-Ala-Leu
Leu-Trp
Ala-Ile
Tyr-Ile
Lys-Thr
Leu-Phe
Leu-Met
Phe-Ile
Ala-Thr
Tyr-Trp
Lys-Val

Val-Pro
Ile-Phe
His-His
Ile-Met
Leu-Ile
Val-Ile
Leu-Leu-Leu
Thr-Asp
Gly-Ile
Gly-Pro
Lys-Ile
Trp-Phe
Thr-Phe
Ile-Val
Trp-Trp
Pro-Tyr
Tyr-Lys
Ile-Pro
Ile-Ala
Leu-D-Leu
Ile-Tyr
Lys-Leu
Arg-Phe
Leu-Leu
Lys-Asp
Val-Tyr-Val

Gly-Gly-D-Leu
Lys-Ser
D-Leu-D-Leu
Leu-Pro
Gly-D-Thr
Lys-Gly
D-Ala-Gly-Gly
Phe-Pro
Phe-ß-Ala
Gly-D-Asp
Gly-D-Ser
D-Leu-Tyr
Pro-Hyp
Gly-D-Ala
ß-Ala-Gly
Lys-Lys
Pro-Phe
Leu-ß-Ala
Met-ß-Ala
ß-Ala-Phe
Negative Control
D-Ala-D-Ala
Negative Control
y-D-Glu-Gly
Pro-Glu
Val-Val

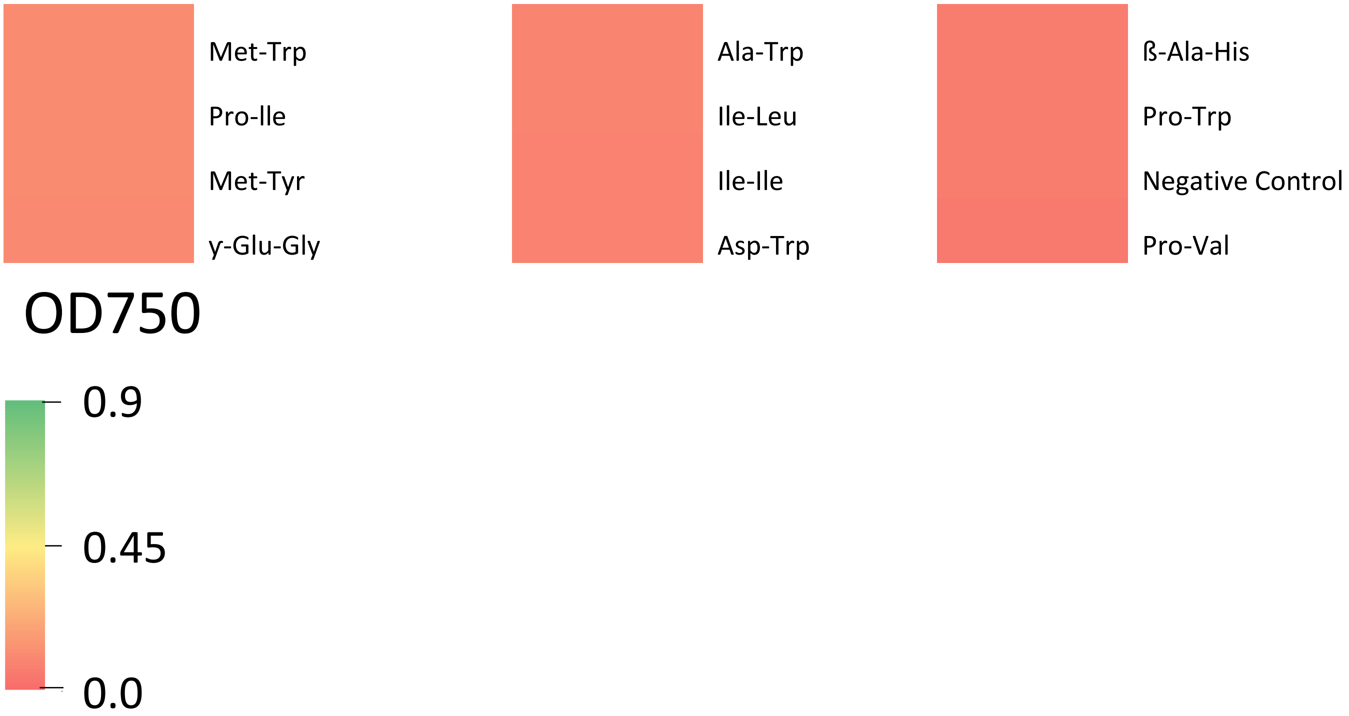


**Supplemental Figure S3.** Nitrogen compounds that supported little to no growth ($OD_{750}$ = 0.0-0.15) by *R. lauricola.* **(A)** Simple nitrogen compounds, and **(B)** peptide nitrogen compounds.

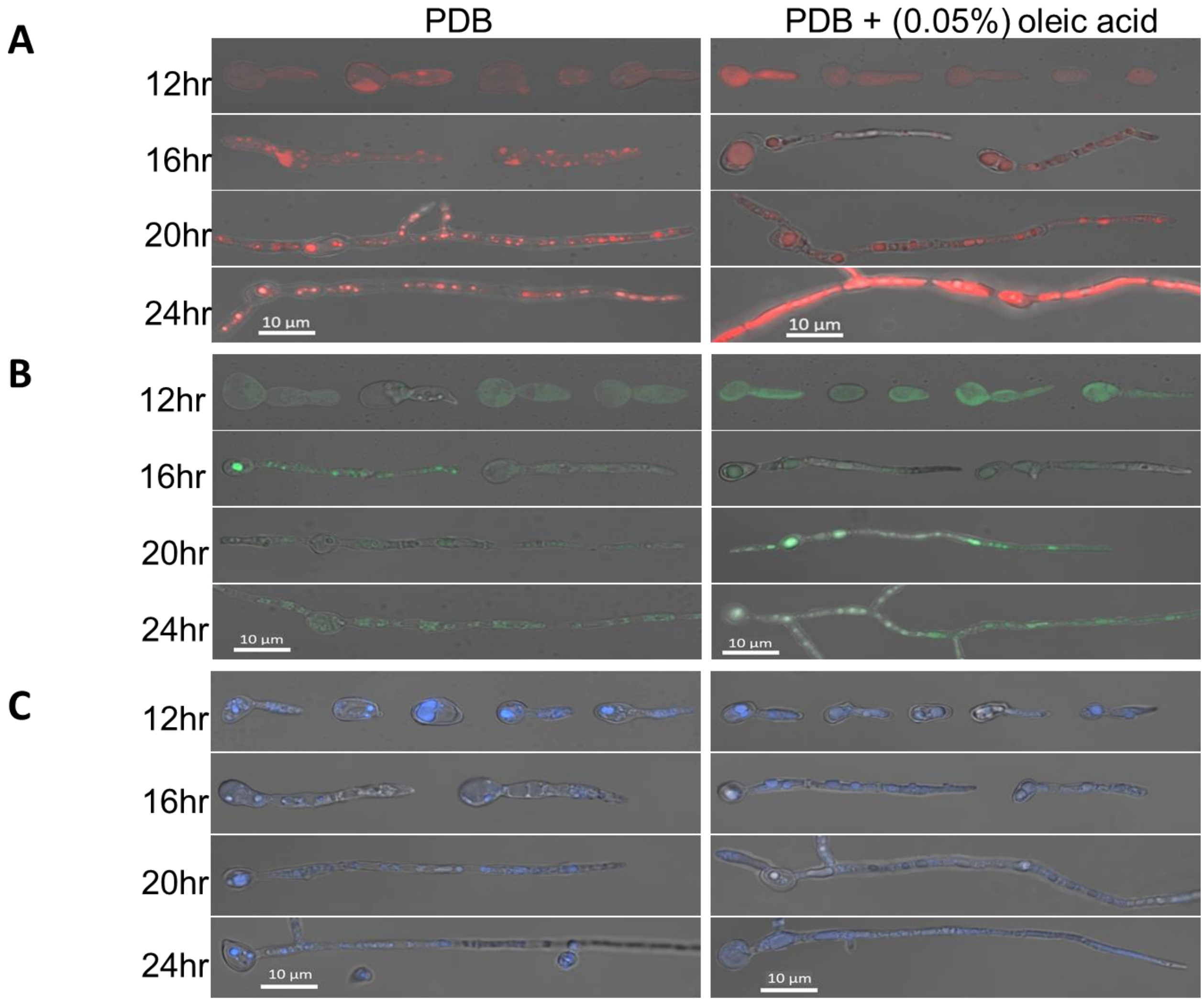


**Supplemental Figure S4.** Assessment of the ability of lipid dyes, Lipi-Red **(A)**, Lipi-Green **(B)**, and Lipi-Blue **(C)** to stain lipid droplets in *R. lauricola*. Cells were grown in PDB (left) or in PDB + 0.05% oleic acid (C18, right), and sampled over a 12-24 h time course.

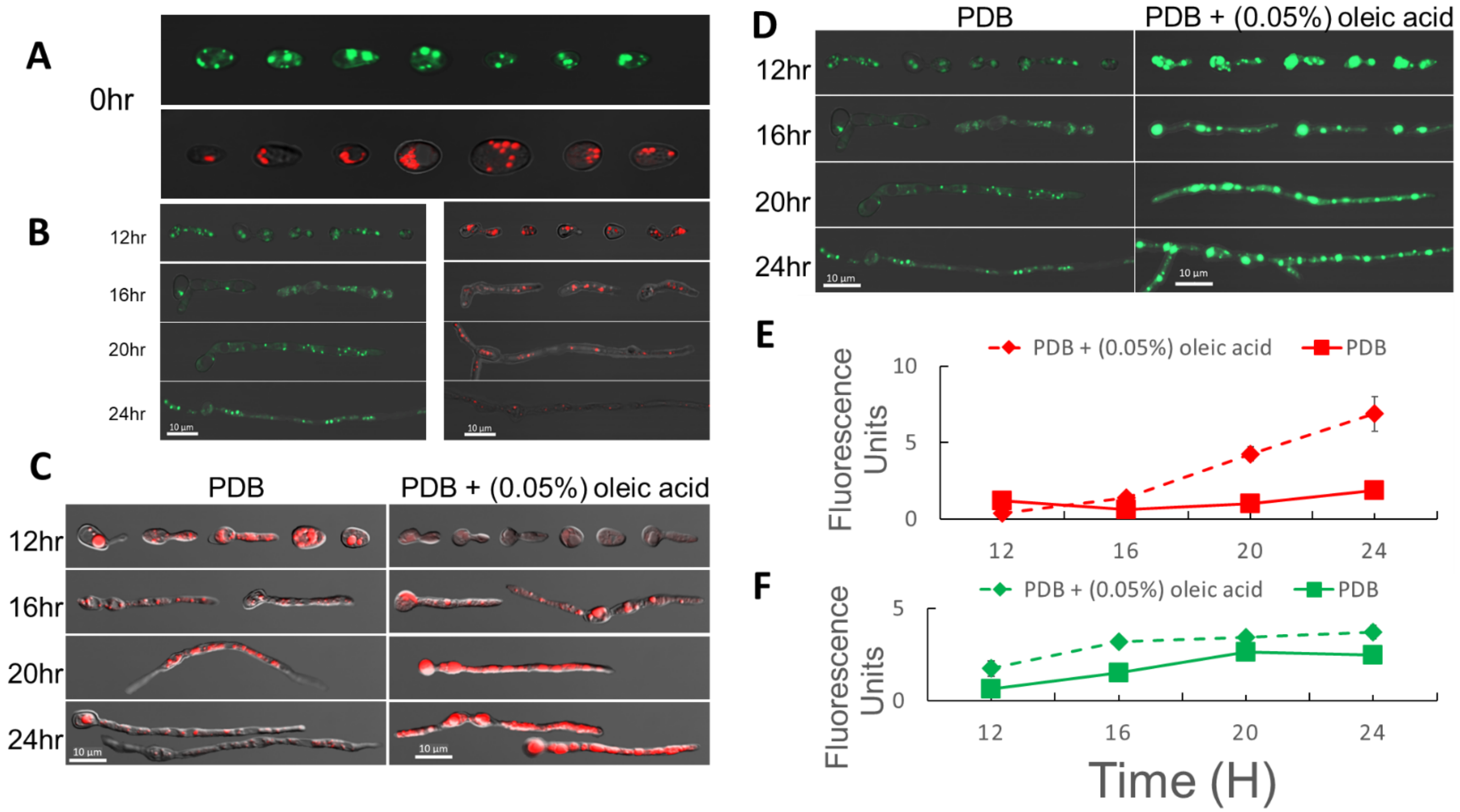


**Supplemental Figure S5.** Representative images of BODIPY and Nile Red staining lipid droplets (LD) in *R. lauricola*. **(A)** *R. lauricola* blastospores collected from PDA plates and immediately stained with BODIPY or Nile Red. **(B)** *R. lauricola* cells grown in PDB and sampled at 12-, 16-, 20-, and 24-h timepoints and stained using BODIPY and Nile Red dyes. **(C)** Nile Red staining comparison between PDB (control) and PDB + C18 (oleic acid) at 12-, 16-, 20-, and 24-h timepoints. (D) BODIPY staining comparison between PDB (control) and PDB + C18 (oleic acid) at 12-, 16-, 20-, and 24-h timepoints. (E) Quantification of Nile Red fluorescence intensity in cells grown under the indicated conditions. (F) Quantification of Nile Red fluorescence intensity in cells grown under the indicated conditions. At least 15-20 images were analyzed in three biological replicates for each time point/dye. Data are shown ± SE.
